# The Effect of Dapagliflozin, a Sodium–Glucose Co-Transporter 2 Inhibitor, on Vancomycin-Induced Nephrotoxicity in Rats

**DOI:** 10.3390/biomedicines13071582

**Published:** 2025-06-27

**Authors:** Seyhmus Tan, Bulent Kaya, Ercan Akburak, Cagri Avci, Kivilcim Eren Ates, Gulfiliz Gonlusen, Tugce Sapmaz Ercakalli, Burak Mete

**Affiliations:** 1 Department of Internal Medicine, Faculty of Medicine, Çukurova University, 01330 Adana, Sarıçam, Turkey; stan@cu.edu.tr (S.T.); drercanakburak@hotmail.com (E.A.); 2 Department of Nephrology, Faculty of Medicine, Çukurova University, 01330 Adana, Sarıçam, Turkey; 3 Department of Virology, Faculty of Ceyhan Veterinary Medicine, Çukurova University, 01930 Adana, Ceyhan, Turkey; cavci@cu.edu.tr; 4 Department of Pathology, Faculty of Medicine, Çukurova University, 01330 Adana, Sarıçam, Turkey; kivilcimerenates@hotmail.com (K.E.A.); gfgonlusen@gmail.com (G.G.); 5 Department of Histology and Embriology, Faculty of Medicine, Çukurova University, 01330 Adana, Sarıçam, Turkey; tsapmaz@cu.edu.tr; 6 Department of Public Health, Faculty of Medicine, Çukurova University, 01330 Adana, Sarıçam, Turkey; burakmete2008@gmail.com

**Keywords:** Vancomycin-induced nephrotoxicity, Dapagliflozin, nephroprotective effect

## Abstract

**Background/Objectives**: Vancomycin-induced nephrotoxicity (VIN) remains a significant clinical challenge, with no effective nephroprotective agent currently established. This study aimed to evaluate the protective effects of the sodium–glucose cotransporter 2 (SGLT2) inhibitor dapagliflozin (DAPA) against VIN in a Wistar albino rat model. **Methods**: Rats were randomly assigned to four groups: control, VA (vancomycin), DAPA (dapagliflozin), and VA+DAPA. Renal function was assessed by measuring serum urea and creatinine. Oxidative stress markers [malondialdehyde (MDA), total oxidant status (TOS), and myeloperoxidase (MPO)], antioxidant enzyme activities [total antioxidant status (TAS), glutathione peroxidase (GPx), catalase (CAT), and superoxide dismutase (SOD)], apoptotic mediators (Bax, Bcl-2, and caspase-3), and pro-inflammatory cytokines [tumor necrosis factor-alpha (TNF-α), interleukin-1 beta (IL-1β), and interleukin-6 (IL-6)] were evaluated. Histopathological and immunohistochemical analyses of kidney tissues were also performed. **Results**: Administration of VA led to significant renal dysfunction, increased oxidative stress, heightened apoptotic activity, and notable histopathological damage. Co-administration of DAPA with VA significantly reduced serum urea and creatinine levels and decreased caspase-3 activity and was associated with a trend toward reduction in both MDA levels and TNF-α expression, as well as the amelioration of histopathological renal injury. However, reductions in IL-1β and IL-6 levels were not statistically significant. Overall, these findings indicate that DAPA exerts nephroprotective effects against VIN by modulating oxidative stress, inflammation, and apoptotic pathways. **Conclusions**: Dapagliflozin may serve as a potential protective agent against vancomycin-induced nephrotoxicity. Further long-term and large-scale clinical studies are warranted to validate these preclinical findings and explore their therapeutic implications.

## 1. Introduction

Vancomycin (VA) is a glycopeptide antibiotic widely used for the treatment of Gram-positive bacterial infections; however, its therapeutic administration requires careful consideration in terms of dosing, administration route, and monitoring due to the risk of nephrotoxicity and the emergence of resistant bacteria [1]. VA preferentially accumulates within the cytoplasm of proximal renal tubular epithelial cells, leading to the potential injury of both these cells and the surrounding interstitium [2]. Several factors—such as pre-existing renal impairment, high daily doses, prolonged therapy, concomitant use of nephrotoxic drugs, and elevated trough levels—are recognized as key risk factors for VA-induced nephrotoxicity (VIN) [3]. Therefore, dose optimization and therapeutic monitoring of VA remain essential, particularly in critically ill patients, in order to balance clinical efficacy with the minimization of nephrotoxicity risk [1].

Although higher target VA levels (15–20 μg/mL) are recommended to combat bacterial resistance and ensure effective treatment, this therapeutic approach is associated with an increased risk of nephrotoxicity [4]. VA-associated acute kidney injury (VA-AKI) typically manifests within 4 to 17 days after the initiation of therapy and is generally reversible upon drug discontinuation [5,6]. However, VA-AKI is linked to prolonged hospitalization, increased hospital readmissions, and higher patient mortality rates [7]. Numerous experimental studies have investigated various pharmacological strategies for the prevention and treatment of VIN [8,9]. Agents such as melatonin, atorvastatin, N-acetylcysteine, vitamins E and C, caffeic acid phenethyl ester, curcumin, Ginkgo biloba, erdosteine, erythropoietin, amrinone, and naringenin have demonstrated the ability to attenuate renal injury in VIN experimental models, primarily via the suppression of oxidative stress and inflammation, and have produced improvements in both histopathological and biochemical parameters [9,10,11,12,13,14,15,16]. Despite promising results in preclinical models and the favorable safety, cost, and availability profiles of these compounds, their routine clinical use cannot yet be recommended due to insufficient evidence from clinical trials [8]. The atrial natriuretic peptide has also demonstrated renoprotective effects in experimental settings—attributed to its enhancement of renal blood flow and anti-inflammatory properties—but its clinical application is hampered by adverse effects, such as hypotension [17]. Importantly, the number of clinical studies remains limited relative to experimental research. Of the few available, a retrospective cohort study indicated the renoprotective effects of melatonin in VA-AKI, whereas a randomized controlled trial highlighted the beneficial impact of N-acetylcysteine in the setting of VIN [18,19]. Additionally, there is some evidence supporting the protective effects of high-dose vitamin C or vitamin E in VIN, though these findings are also limited to a minority of clinical investigations [20,21]. Conventional antioxidants fail to adequately address all the pathophysiological mechanisms involved in multifactorial renal injury, underscoring the necessity for combination therapies that target multiple pathways in AKI [22]. Consequently, there is increasing interest in developing novel renoprotective agents—such as sodium–glucose co-transporter 2 (SGLT2) inhibitors with proven clinical efficacy—that can target this complex multifactorial pathogenesis [23].

SGLT2 inhibitors are a novel class of antidiabetic agents that decrease sodium and glucose reabsorption in the renal proximal tubules [24]. In addition to their established antidiabetic actions, SGLT2 inhibitors exhibit notable antioxidative and anti-inflammatory properties [25,26]. In diabetic individuals, these agents have been shown to slow the decline of the glomerular filtration rate (GFR), reduce proteinuria progression, and confer significant renoprotective effects [25]. The favorable outcomes observed with SGLT2 inhibitors in diabetic nephropathy have prompted investigation into their potential benefits in various organ systems—including the kidneys, heart, and liver—even beyond populations with diabetes [27,28,29,30,31]. Accordingly, their renoprotective potential has been evaluated in several animal models of drug-induced nephrotoxicity, including those induced by gentamicin, cyclosporin, and colistin [32,33,34,35,36]. Furthermore, SGLT2 inhibitors have been shown to mitigate reperfusion injury following renal ischemia in mouse models [37]. However, no renoprotective effect was observed in mice with oxalate-induced nephrocalcinosis or in rats following subtotal (5/6) nephrectomy [38,39]. Recently, it was reported that the SGLT2 inhibitor dapagliflozin (DAPA) ameliorated apoptosis, oxidative stress, and renal histopathological findings in VIN, thereby improving AKI [40]. Notably, these effects were attributed primarily to improvements in the antioxidative and anti-apoptotic parameters [40]. In addition to oxidative stress and apoptosis, inflammation has been implicated as a key contributor to renal injury in VIN [41].

In our present study, we aimed to evaluate the involvement of inflammation—alongside apoptosis and oxidative stress—in the VIN model, as well as to assess the anti-inflammatory effects of the SGLT2 inhibitor DAPA on VIN. To this end, we designed an in-depth experimental approach assessing not only oxidative stress and apoptosis parameters but also inflammatory markers and the corresponding renal histopathological changes associated with these inflammatory parameters.

## 2. Materials and Methods

### 2.1. Experimental Rats

Two-month-old male Wistar albino rats (*n* = 28) weighing 250–350 g were used in this study. The animals were obtained from the Experimental Animal Laboratories of Çukurova University Faculty of Medicine. Prior to the experiment, the rats were housed under standard laboratory conditions: a constant room temperature of 23 ± 2 °C, relative humidity of 60 ± 5%, a 12 h light/dark cycle, and free access to standard rat chow (Feed Institution Standard Rat Food) and water. No special diet was provided. After a one-week acclimatization period, the rats were randomly divided into four groups of seven animals each for the experimental study. All procedures were performed in accordance with the Guide for the Care and Use of Laboratory Animals [42].

### 2.2. Ethics Committee

This study was approved by the Animal Studies Ethics Committee of our university (Approval number: 20.07.2023/5). All procedures were conducted in accordance with the guidelines set forth in the Guide for the Care and Use of Laboratory Animals.

### 2.3. Drugs, Rationale for Dose Selection, Administration Route, and Duration

The duration of treatment, administration routes, and dosing regimen in this study were determined based on both pharmacokinetic data and previous experimental studies investigating vancomycin- and dapagliflozin-associated renal injury in rats [43]. A 7-day treatment period was chosen, as this duration is widely used and shown to be sufficient for inducing renal injury with vancomycin while allowing for the meaningful assessment of the potential protective effects of co-administered agents without excessive mortality [44].

The intraperitoneal (IP) route was selected for VA since the drug has poor oral bioavailability, and IP administration ensures consistent systemic absorption and reproducible nephrotoxic effects; this protocol is also supported by numerous rodent studies [45]. DAPA was given by oral gavage, in line with its clinical use in humans, and to achieve effective gastrointestinal absorption, as demonstrated in previous experimental reports [34,37].

DAPA (Forziga^®^ oral tablets) was obtained from AstraZeneca Pharmaceuticals LP (Wilmington, DE, USA). The intravenous formulation of VA was procured from Koçak Farma Pharmaceutical and Chemical Industry Inc. (Istanbul, Turkey).

### 2.4. Animals and Experimental Groups

Male Wistar albino rats (*n* = 28) were divided into four equal groups at random and received the following treatments:

- Group 1 (Control): Rats received an IP injection of saline for seven days.
- Group 2 (VA): Rats received VA (200 mg/kg) via an IP injection twice daily for seven days, as described in previous studies [9,46].
- Group 3 (DAPA): Rats received DAPA (10 mg/kg) by oral gavage for seven consecutive days, as described in previous studies [34,37].
- Group 4 (VA+DAPA): Rats received DAPA (10 mg/kg) by oral gavage for seven days and VA (200 mg/kg) via an IP injection twice daily for the same period.

### 2.5. Operation Procedures and Measurements

All procedures were performed in accordance with standard protocols for animal experimentation, as described in the Guide for the Care and Use of Laboratory Animals [42]. The duration of the experiment was set to 7 days. All procedures were performed between 09:00 and 10:00 a.m. to minimize circadian variation. Injections were administered twice daily at 12 h intervals using an insulin syringe into the left lower quadrant of each rat to ensure consistent dosing volumes. Aspiration was performed before each injection to prevent inadvertent intravenous administration. This injection protocol was continued for seven days.

On day eight, anesthesia was induced via an IP injection of ketamine (50 mg/kg) and xylazine (10 mg/kg). Blood samples were collected by intracardiac puncture. The samples were centrifuged at 3000 rpm for 10 min to separate the serum, which was then stored at –80 °C until analysis. The rats were sacrificed by cervical dislocation. Immediately following sacrifice, kidney tissues were resected and placed in 10% neutral-buffered formalin for histopathological evaluation.

On the day of analysis, oxidative stress markers [malondialdehyde (MDA), total oxidant status (TOS), and myeloperoxidase (MPO)], antioxidant markers [total antioxidant status (TAS), glutathione peroxidase (GPx), catalase (CAT), and superoxide dismutase (SOD)], pro-apoptotic mediators [Bcl-2-associated X protein (Bax) and caspase-3 (Casp-3)], an anti-apoptotic mediator [B-cell lymphoma-2 (Bcl-2)], and renal function parameters (urea and creatinine) were measured in serum samples thawed at room temperature. All histological, immunohistochemical, and biochemical analyses were performed by investigators blinded to group allocation in order to minimize bias.

#### 2.5.1. Measurement of Serum Urea and Creatinine Levels

Serum urea and creatinine levels were determined by a colorimetric method using an autoanalyzer (Mindray-BS400, Foshan, China) and commercial kits (Otto Scientific, Turkey; cat. no.: OttoBC157 for urea and OttoBC139 for creatinine). Creatinine was measured in micromoles per liter (µmol/L) and urea in milligrams per deciliter (mg/dL). The results are expressed as mg/dL. Urea values were divided by 2.14 to obtain blood urea nitrogen (BUN).

#### 2.5.2. Measurement of MDA and CAT Levels

Serum MDA levels were determined colorimetrically using a commercial kit (Otto Scientific, Turkey; cat. no.: Otto1001) in accordance with the manufacturer’s instructions, based on the method described by Yoshioka et al. [47]. CAT activity was measured colorimetrically with a kit (Elabscience, USA; cat. no.: E-BC-K031-S), following the standard procedure reported by Aebi [48]. Both analyses were performed using a colorimetric method (Rel Assay Diagnostics, Turkey). MDA levels are expressed as nanomoles per gram (nmol/g) and CAT activity as units per liter (U/L).

#### 2.5.3. Measurement of Bax, Bcl-2, and Caspase-3 Levels

Serum Casp-3 levels were measured using a competitive ELISA kit (Elabscience, Houston, TX, USA; cat. no.: E-EL-R0160), according to the manufacturer’s instructions, based on the method originally described by Engvall and Perlmann [49]. The colorimetric reactions were measured at 450 nm using a microplate reader (BIO-TEK EL X 800, Agilent Technologies, Santa Clara, CA, USA). Washing steps were performed with an automatic strip washer (BIO-TEK EL X 50, Agilent Technologies, Santa Clara, CA, USA). The results were calculated from the standard curve.

Serum Bax and Bcl-2 levels were measured using ELISA kits (BT-Lab, Shanghai, China; cat. no.: E0034Ra for Bax and E0037Ra for Bcl-2), based on the method originally described by Engvall and Perlmann [49]. The colorimetric reactions were read at 450 nm with a microplate reader (BIO-TEK EL X 800, USA), and washing steps were performed using an automatic strip washer (BIO-TEK EL X 50, USA). Casp-3, Bax, and Bcl-2 concentrations are expressed as nanograms per milliliter (ng/mL).

#### 2.5.4. Measurement of SOD, GPx, and MPO Levels

Serum levels of SOD, GPx, and MPO were determined colorimetrically using commercial kits (Otto Scientific, Ankara, Turkey; cat. no.: Otto3047 for SOD, Otto2085 for GPx, Otto3048 for MPO) with a Mindray-BS400 autoanalyzer (Mindray, Shenzhen, China), according to the manufacturer’s instructions. SOD activity was measured based on the method described by Marklund and Marklund [50], GPx activity according to Paglia and Valentine [51], and MPO activity as described by Bradley et al. [50]. GPx and MPO levels are expressed as U/L and SOD activity as U/mL.

#### 2.5.5. Measurement of TAS and TOS Levels

Serum TAS and TOS levels were measured colorimetrically using commercial kits (Rel Assay Diagnostics, Gaziantep, Turkey; cat. no.: RL0017 for TAS and RL0024 for TOS) with a Mindray-BS400 autoanalyzer (China), according to the manufacturer’s instructions, based on the methods described by Erel [52,53]. The TAS results are expressed as mmol/L and the TOS results as μmol H_2_O_2_ equivalents/L.

#### 2.5.6. Immunohistochemical Evaluation of TNF-α, IL-1β, and IL-6 in Kidney Tissues

Rat kidney tissues were incubated with primary antibodies against tumor necrosis factor-alpha (TNF-α), interleukin-6 (IL-6), and interleukin-1 beta (IL-1β) (all from Santa Cruz Biotechnology, Dallas, TX, USA; 1:100 dilution) for 60 min each. Antigen retrieval was carried out using the 20 min ER2 standard protocol with LEICA/Bond Epitope Retrieval 2 RTU. Immunohistochemical staining was carried out using the standard protocol [54]. For secondary detection, a standard immunohistochemical protocol was employed using the DS9800 LEICA/Bond Polymer Refine Detection Kit (Leica Biosystems, Newcastle upon Tyne, UK). Immunohistochemical staining was evaluated for each group by assessing five distinct areas in three sections per sample. Staining intensity was scored semi-quantitatively from 0 to 3 (0 = negative, 1 = weak, 2 = moderate, and 3 = strong), modified from the original scoring system of light (+), moderate (++), severe (+++), and very severe (++++), as previously described [55]. All evaluations were performed by a pathologist, who was blinded to the experimental groups, using an Olympus BX51 light microscope (Olympus GmbH, Tokyo, Japan).

### 2.6. Histopathological Examination of Renal Tissues

Renal tissues from all groups were fixed in 10% neutral-buffered formalin, dehydrated in ethanol, cleared with xylene, and embedded in paraffin. Sections of 5 μm thickness were obtained from the paraffin-embedded renal blocks and stained with hematoxylin and eosin (H&E) and periodic acid–Schiff (PAS). Histological evaluation was performed by a pathologist, who was blinded to the groups, using an Olympus BX51 (Olympus GmbH, Tokyo, Japan) light microscope. Renal tissue samples were examined by light microscopy for the presence of tubular necrosis, tubular vacuolization, tubular dilatation, tubular atrophy, tubular desquamation, mononuclear cell infiltration in the medulla, necrosis in the medulla, necrosis in the cortex, inflammation, and hyaline casts.

The degree of damage was scored as follows: (–) indicates no significant histopathological damage; (+) indicates mild damage; (++) indicates moderate damage; and (+++) indicates severe damage, as previously described [56].

### 2.7. Statistical Analyses

Data were statistically analyzed using SPSS version 27.0 (IBM, Armonk, NY, USA) and are presented as mean ± standard deviation (SD). The normality of distribution for each parameter was assessed using the Shapiro–Wilk test. For normally distributed data, comparisons among rat groups were performed using one-way analysis of variance (ANOVA) followed by the Bonferroni post-hoc test. For non-normally distributed data, the Kruskal–Wallis test was applied, and post-hoc pairwise group comparisons were determined using Dunn’s test with Bonferroni correction. A *p*-value less than 0.05 was considered statistically significant in all analyses.

## 3. Results

Table 1 presents a comparative summary of renal function tests, oxidative stress markers (MDA, TOS, and MPO), antioxidant system parameters (TAS, GPx, CAT, and SOD), pro-apoptotic mediators (Bax and Casp-3), the anti-apoptotic mediator (Bcl-2), and renal function test parameters (urea and creatinine) among four groups of rats (control, DAPA, VA, and VA+DAPA). Co-administration of DAPA with VA significantly reduced serum urea and creatinine levels and decreased caspase-3 activity and was associated with a trend toward a reduction in MDA levels. SOD levels were significantly higher in the VA and VA+DAPA groups compared to the controls. No significant differences were observed among the groups regarding TOS, MPO, GPx, CAT, Bax, or Bcl-2 levels, except for a significant increase in TAS in the VA group. These findings suggest that VA impairs renal function and increases oxidative and apoptotic parameters, whereas DAPA treatment partially ameliorates these detrimental effects.

### 3.1. Serum Urea and Creatinine Levels

**Figure 1:**
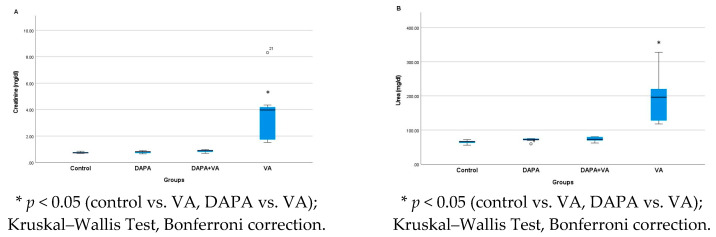
(**A**) A comparison of the serum creatinine levels and (**B**) serum urea levels among the experimental groups. Vancomycin (VA) markedly increased creatinine and urea, indicating significant renal dysfunction. Co-administration of dapagliflozin (VA+DAPA) reduced these levels close to the control group, suggesting a protective effect. No significant changes were observed in the control or DAPA-only groups.

A significant difference was observed in the serum urea and creatinine levels among the groups (*p* < 0.001). The VA group showed the highest serum urea and creatinine levels, indicating marked renal dysfunction. In contrast, the DAPA+VA group had significantly lower levels of both markers compared to the VA group (*p* < 0.001 for both parameters), suggesting a protective effect of dapagliflozin. No significant elevations were observed in the control or DAPA-only groups. These findings demonstrate that vancomycin induces renal impairment, while dapagliflozin co-administration alleviates this effect (Figure 1A,B).

### 3.2. MDA, TOS, and MPO Levels

**Figure 2:**
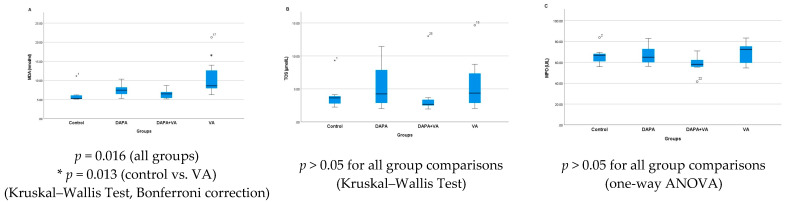
(**A**) The serum levels of malondialdehyde (MDA), (**B**) total oxidant status (TOS), and (**C**) myeloperoxidase (MPO) across the groups. The VA group exhibited significantly elevated MDA, reflecting increased oxidative stress. The addition of dapagliflozin showed a reduction trend but did not reach significance. No significant changes were detected in TOS or MPO among the groups.

In the study, significant differences were found among the four groups in terms of the MDA, TOS, and MPO levels. MDA levels showed a statistically significant difference between the groups (Kruskal–Wallis, *p* = 0.016). In particular, the VA group had markedly higher MDA levels compared to the control group (*p* = 0.013). Although the addition of DAPA to the VA group (the DAPA+VA group) resulted in a decreasing trend in MDA levels compared to the VA group, this difference was not statistically significant (Figure 2A). There were no significant differences among the groups in terms of TOS (*p* = 0.609) or MPO levels (ANOVA, *p* = 0.205). In summary, while a clear increase in MDA levels was observed, especially in the VA group—with a trend toward reduction with additional DAPA—TOS and MPO levels did not show any significant changes among the groups (Figure 2B,C).

### 3.3. TAS, GPx, CAT, and SOD Levels

**Figure 3:**
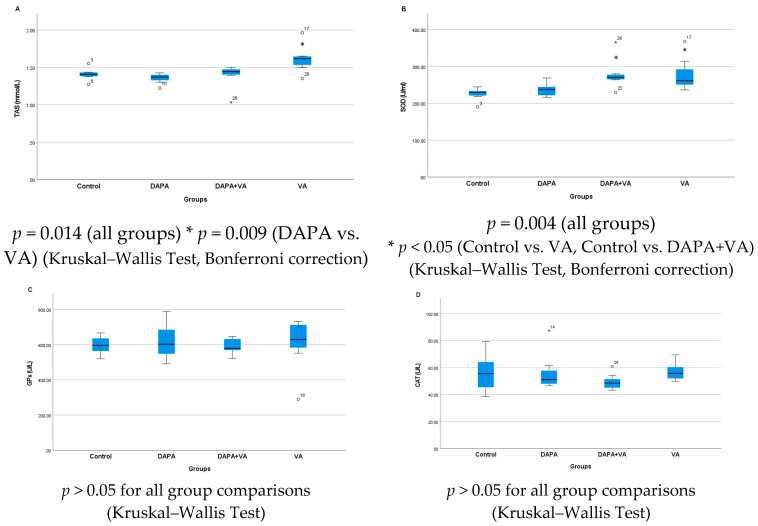
The group comparisons of (**A**) total antioxidant status (TAS), (**B**) superoxide dismutase (SOD), (**C**) glutathione peroxidase (GPx), and (**D**) catalase (CAT) in the serum. The VA group showed higher TAS and SOD levels than the control and DAPA groups. Co-administration of DAPA with VA did not significantly alter these parameters. GPx and CAT levels did not differ significantly among groups.

Serum TAS levels showed a significant difference among the groups (*p* = 0.014). Multiple comparisons reveal that serum TAS levels in the VA group were significantly higher than those in both the control and DAPA groups. The combined administration of VA and DAPA did not result in a significant change in TAS levels. Similarly, serum SOD levels differed significantly between groups (*p* = 0.004). SOD levels were notably higher in the VA and DAPA+VA groups compared to the control and DAPA-only groups. The co-administration of VA and DAPA did not lead to a significant reduction in SOD levels. No significant differences were observed among the groups for serum GPx and CAT levels (*p* > 0.05 for both parameters). In conclusion, VA treatment resulted in increased serum TAS and SOD levels without affecting GPx and CAT levels, while DAPA treatment alone did not have a significant impact on these parameters (Figure 3A–D).

### 3.4. Bax, Bcl-2, and Casp-3 Enzyme Activities

**Figure 4:**
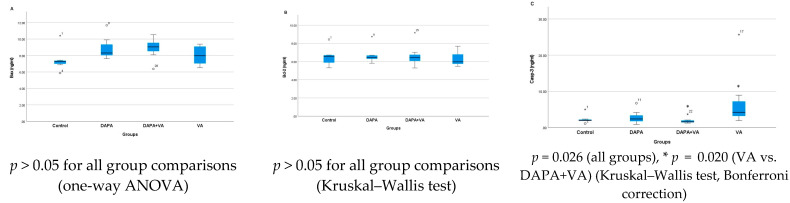
Group-wise comparison of (**A**) Bax, (**B**) Bcl-2, and (**C**) Caspase-3 activities. Vancomycin administration increased caspase-3 activity, indicating apoptosis, while co-administration of dapagliflozin significantly reduced this effect. Bax and Bcl-2 levels showed no significant difference among groups.

A significant difference was observed in Casp-3 activity among the groups (*p* = 0.026). VA administration increased Casp-3 activity, whereas co-administration of DAPA with VA significantly attenuated this increase (*p* = 0.020). No significant differences were found among the groups in terms of Bax or Bcl-2 levels (*p* > 0.05 for all comparisons). In conclusion, VA increases Casp-3 activity, but this effect is mitigated by DAPA co-administration, while no notable changes were observed in Bax and Bcl-2 levels (Figure 4A–C).

### 3.5. Histopathological Studies

**Figure 5:**
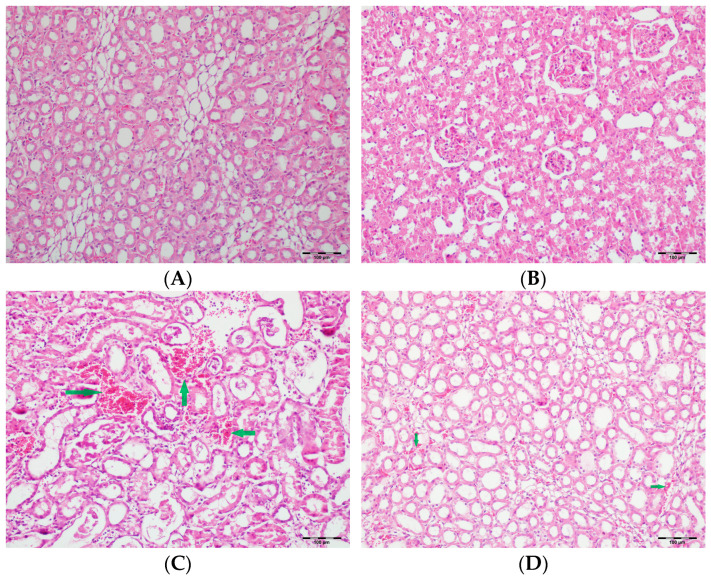
(**A**) Control group; (**B**) DAPA group; (**C**) VA group; (**D**) VA+DAPA group (H&E, ×100). Representative kidney sections showing medullary hemorrhage (green arrows) in different groups. Severe hemorrhage was observed in VA group, indicating vancomycin-induced injury, whereas the VA+DAPA group showed substantial reduction in damage. Control and DAPA groups displayed normal histology.

**Figure 6:**
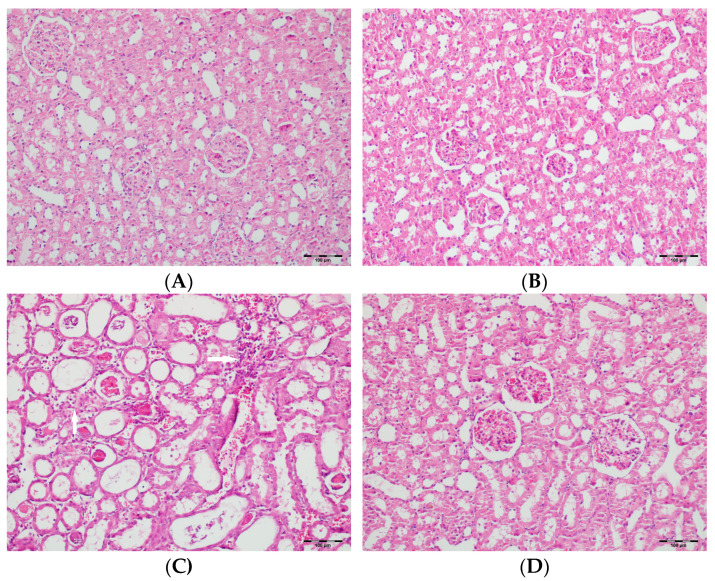
(**A**) Control group; (**B**) DAPA group; (**C**) VA group; (**D**) VA+DAPA group (H&E, ×100). Representative histological sections highlighting mononuclear cell infiltration (white arrows). The most prominent infiltration was seen in VA group; VA+DAPA showed reduction, while control and DAPA had nearly normal appearances. Mononuclear cell infiltration in kidney sections is indicated by white arrows in all groups.

**Figure 7:**
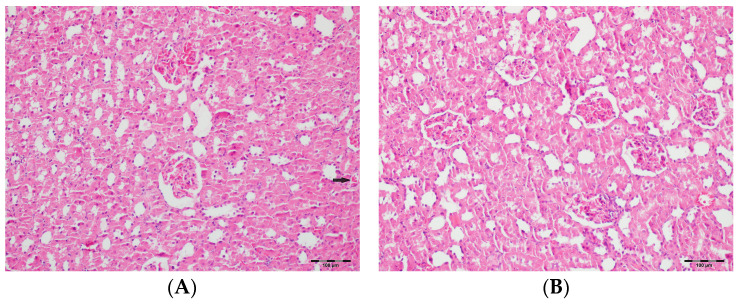

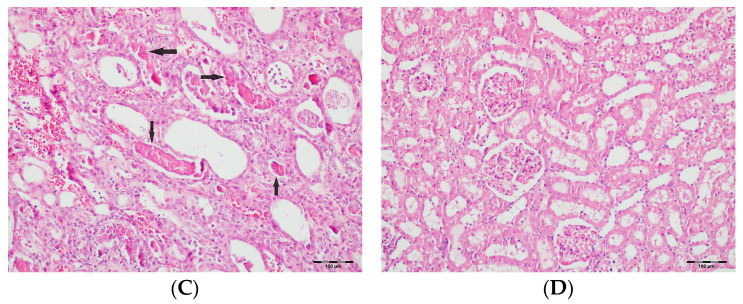
(**A**) Control group; (**B**) DAPA group; (**C**) VA group; (**D**) VA+DAPA group (H&E, ×100). Representative kidney tissue images showing tubular desquamation (black arrows) in different groups. Marked tubular epithelial cell desquamation was most prominent in the VA group, indicating severe tubular injury. The VA+DAPA group exhibited a significant reduction in desquamation, suggesting a protective effect of dapagliflozin. The control and DAPA-only groups displayed normal tubular epithelium with no significant desquamation.

**Figure 8:**
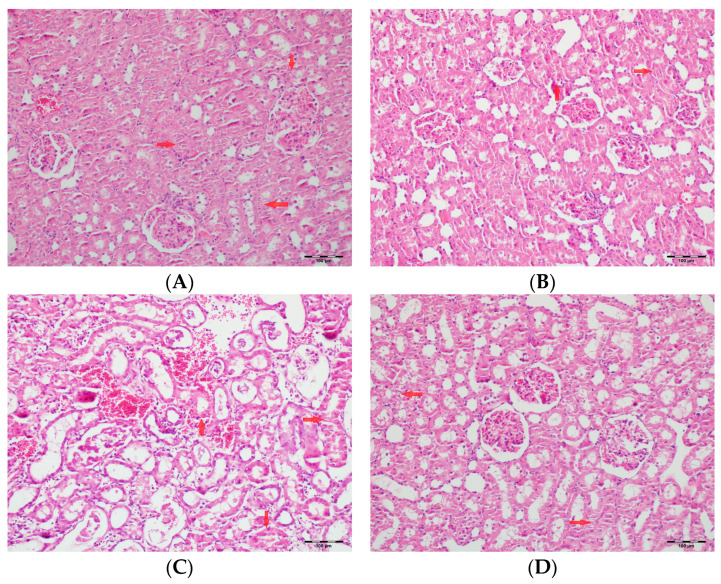
(**A**) Control group; (**B**) DAPA group; (**C**) VA group; (**D**) VA+DAPA group (H&E, ×100). Representative kidney tissue sections demonstrating tubular necrosis (red arrows) in each group. Extensive tubular necrosis—characterized by loss of tubular epithelial cells and fragmentation—was most prominent in the VA group, reflecting severe nephrotoxicity. In the VA+DAPA group, necrosis was significantly reduced, suggesting a protective role of dapagliflozin. The control and DAPA-only groups showed normal tubular architecture without necrosis.

**Figure 9:**
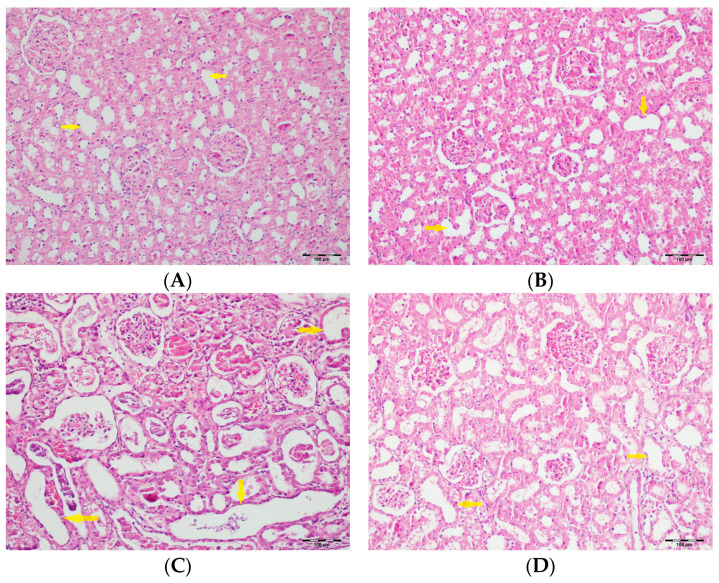
(**A**) Control group; (**B**) DAPA group; (**C**) VA group; (**D**) VA+DAPA group (H&E, ×100). Representative micrographs illustrating tubular dilatation (yellow arrows) in the different experimental groups. The VA group exhibited pronounced dilatation of renal tubules, reflecting acute tubular injury secondary to vancomycin exposure. This pathological finding was significantly reduced in the VA+DAPA group, indicating the protective effect of dapagliflozin. The control and DAPA-only groups showed normal tubular morphology without dilatation.

**Figure 10:**
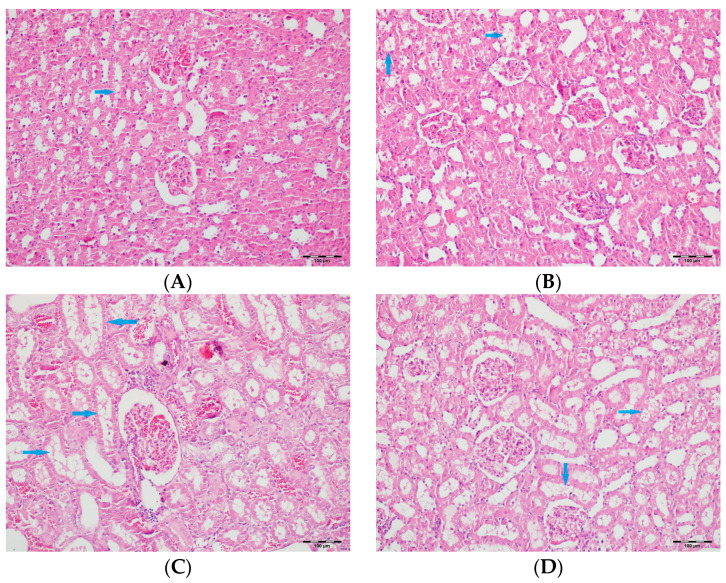
(**A**) Control group; (**B**) DAPA group; (**C**) VA group; (**D**) VA+DAPA group (H&E, ×100). Sections showing tubular vacuolization (blue arrows) and glomerular changes in each group. The VA group displayed marked tubular vacuolization, glomerular congestion, mesangial expansion, and partial sclerosis, all indicative of vancomycin-induced tubular and glomerular injury. These pathological alterations were less severe in the VA+DAPA group, demonstrating the ameliorative effect of dapagliflozin. The control and DAPA-only groups had normal glomerular morphology without significant changes.

**Figure 11:**
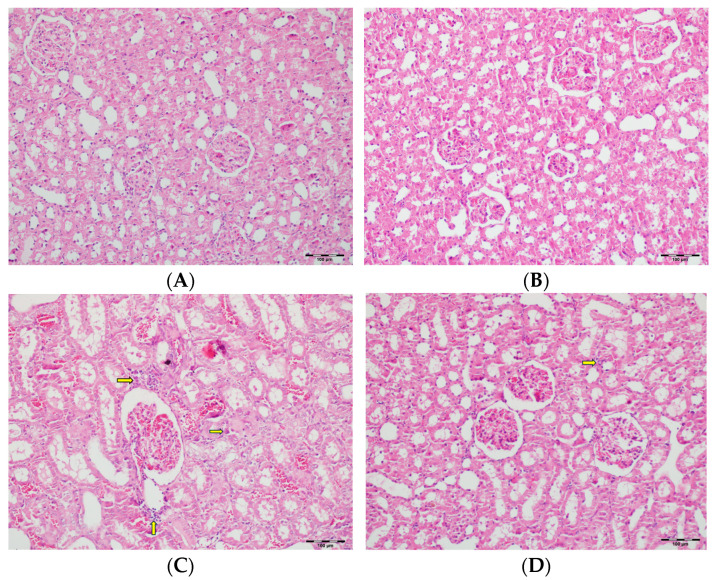
(**A**) Control group; (**B**) DAPA group; (**C**) VA group; (**D**) VA+DAPA group (H&E, ×100). Representative kidney tissue sections demonstrating tubular inflammation (yellow arrows) in each group. The VA group exhibited marked infiltration of inflammatory cells into and around renal tubules, indicating significant tubular inflammation and acute tissue injury. The extent and severity of inflammation were substantially reduced in the VA+DAPA group, suggesting the anti-inflammatory effect of dapagliflozin. The control and DAPA-only groups showed normal tubular appearance without evidence of inflammation.

**Figure 12:**
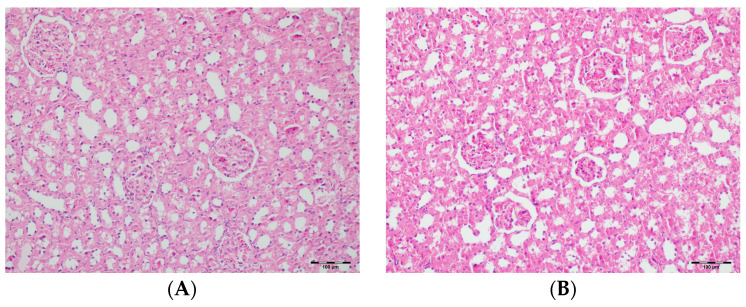

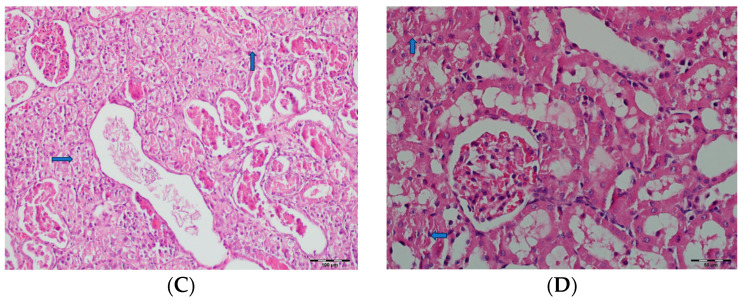
(**A**) Control group; (**B**) DAPA group; (**C**) VA group; (**D**) VA+DAPA group (H&E, ×100). Representative kidney cortex sections showing areas of necrosis (blue arrows) in each group. Extensive cortical necrosis was especially prominent in the VA group, indicating severe renal injury. In the VA+DAPA group, cortical necrosis was substantially reduced, demonstrating the protective effect of dapagliflozin. The control and DAPA-only groups showed normal cortical structures without evidence of necrosis.

**Figure 13:**
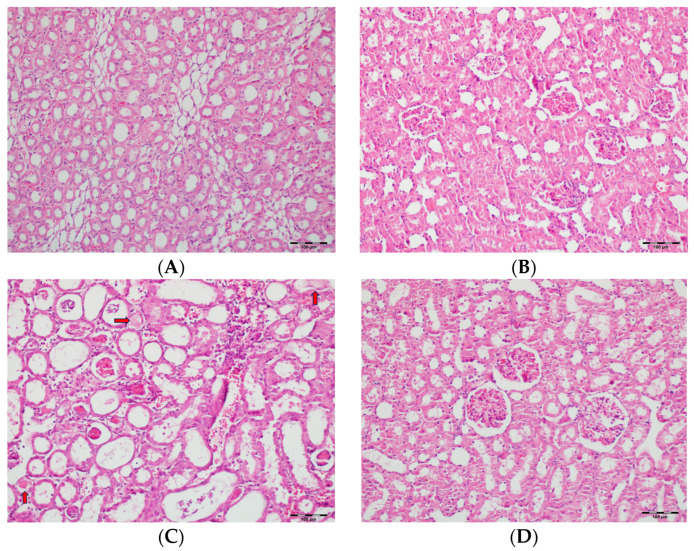
(**A**) Control group; (**B**) DAPA group; (**C**) VA group; (**D**) VA+DAPA group (H&E, ×100). Representative kidney sections showing hyaline casts (red arrows) within renal tubules in each group. Extensive accumulation of hyaline casts was observed in the VA group, reflecting acute tubular injury. In the VA+DAPA group, the presence of hyaline casts was notably reduced, indicating the protective effect of dapagliflozin. The control and DAPA-only groups showed minimal or no hyaline casts in renal tubules.

Table 2 summarizes the renal histopathological findings among four experimental groups of rats (control, DAPA, VA, and VA+DAPA). In the VA group, significant increases were observed in multiple indicators of renal injury—including hyaline casts, tubular necrosis, tubular dilatation, tubular vacuolization, tubular atrophy, tubular inflammation, interstitial edema, medullary hemorrhage, cortical necrotic areas, and tubular desquamation—compared to the other groups. Co-administration of DAPA with VA resulted in a significant reduction in all these pathological changes (*p* < 0.001). The control and DAPA groups generally maintained normal renal histology without significant damage. These findings indicate that VA induces marked renal injury, whereas DAPA treatment considerably mitigates these adverse histopathological changes (Figure 5, Figure 6, Figure 7, Figure 8, Figure 9, Figure 10, Figure 11, Figure 12 and Figure 13).

### 3.6. Immunohistochemical Evaluation of IL-1β, IL-6, and TNF-α in Kidney Tissue

According to Table 3, immunohistochemical analysis reveals significant differences in the renal tissue immunostaining intensities of TNF-α, IL-1β, and IL-6 among the rat groups (*p* < 0.001 for all parameters). In the VA group, immunostaining levels of TNF-α, IL-1β, and IL-6 were markedly increased compared to both the control and DAPA-only groups. In the group receiving both VA and DAPA, the most prominent reduction in immunostaining scores was observed for TNF-α, whereas IL-1β and IL-6 levels remained elevated but were lower than those in the VA group. In both the control and DAPA-only groups, TNF-α, IL-1β, and IL-6 levels were low and comparable. These findings indicate that VA administration induces a strong inflammatory response in renal tissue, and co-administration of DAPA is associated with a more pronounced trend toward a reduction in TNF-α expression, while its effects on IL-1β and IL-6 show a more limited trend of decrease.

**Figure 14:**
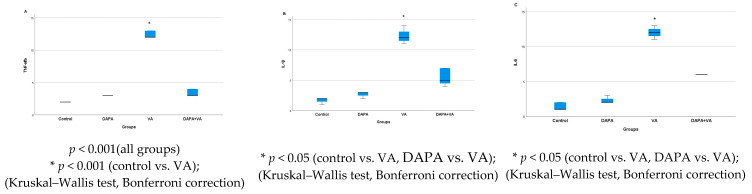
(**A**) The immunostaining intensities of TNF-α by group; (**B**) the immunostaining intensities of IL-1β by group; (**C**) the immunostaining intensities of IL-6 by group. In the VA group, the expression levels of all three pro-inflammatory cytokines were markedly increased, indicating enhanced renal inflammation. In the VA+DAPA group, immunostaining intensities for TNF-α, IL-1β, and IL-6 were significantly reduced, suggesting that dapagliflozin attenuates the inflammatory response. The control and DAPA-only groups showed minimal cytokine expression, consistent with normal renal tissue.

**Figure 15:**
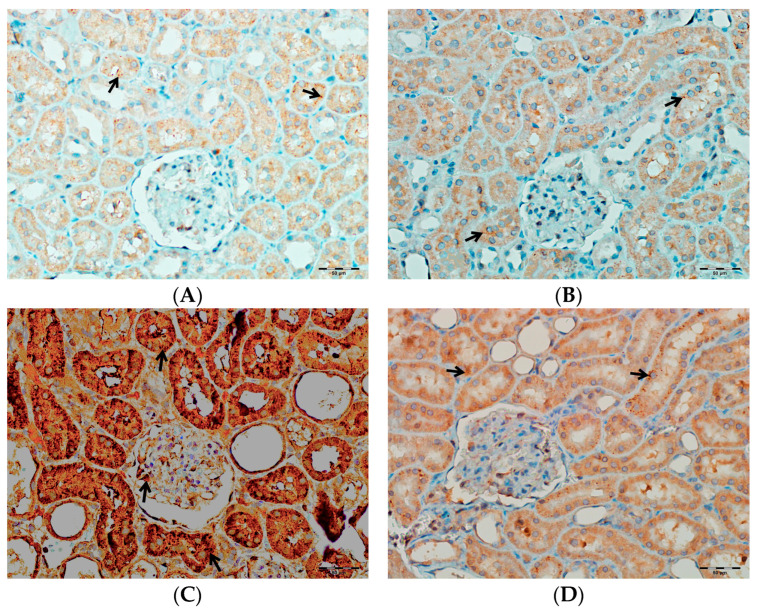
(**A**) Control groups; (**B**) DAPA groups; (**C**) VA groups; (**D**) VA+DAPA groups. The immunostaining intensities of TNF-α in all groups. TNF-α immunohistochemical staining is indicated by the black arrow. The VA group (**C**) showed intense TNF-α staining, reflecting significant renal inflammation. In the VA+DAPA group (**D**), TNF-α expression was markedly reduced, indicating the anti-inflammatory effect of dapagliflozin. The control (**A**) and DAPA groups (**B**) displayed minimal or no TNF-α staining.

**Figure 16:**
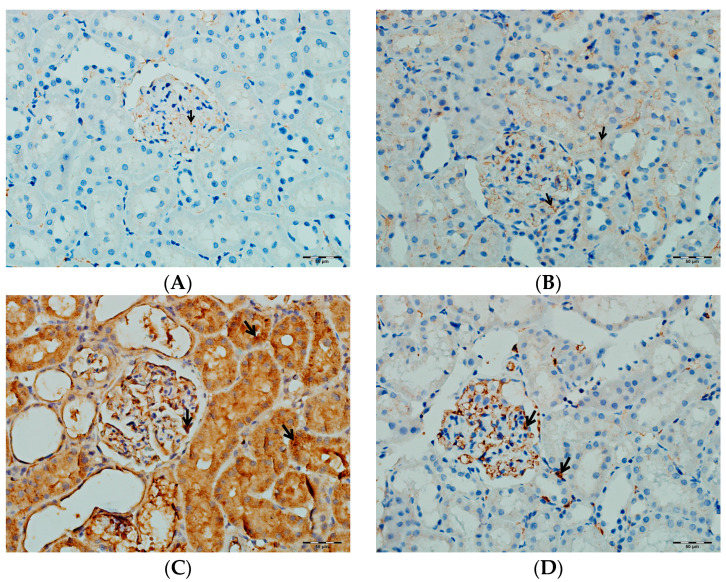
(**A**) Control groups; (**B**) DAPA groups; (**C**) VA groups; (**D**) VA+DAPA groups. The immunostaining intensities of IL-1β in all groups. IL-1β immunohistochemical staining is indicated by the black arrow. The VA group (**C**) showed intense IL-1β staining, indicating pronounced renal inflammation. In the VA+DAPA group (**D**), IL-1β immunostaining was markedly reduced, suggesting that dapagliflozin attenuates the inflammatory response. The control (**A**) and DAPA groups (**B**) displayed minimal or no IL-1β staining.

**Figure 17:**
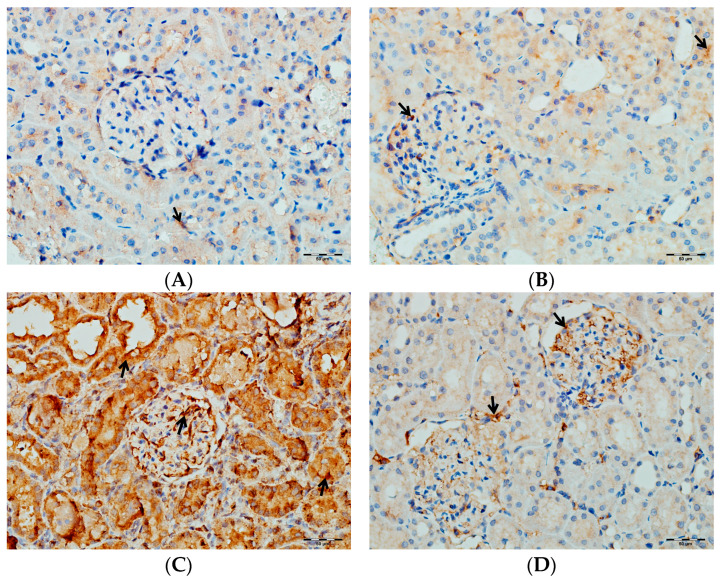
(**A**) Control groups; (**B**) DAPA groups; (**C**) VA groups; (**D**) VA+DAPA groups. The immunostaining intensities of IL-6 in all groups. IL-6 immunohistochemical staining is indicated by the black arrow. The VA group (**C**) showed intense IL-6 staining, reflecting enhanced renal inflammation. In the VA+DAPA group (**D**), IL-6 immunostaining was markedly reduced, demonstrating the anti-inflammatory effect of dapagliflozin. The control (**A**) and DAPA groups (**B**) displayed minimal or no IL-6 staining.

As shown in Figure 14, immunohistochemical staining scores for TNF-α, IL-1β, and IL-6 were significantly higher in the VA group compared to the control group (*p* < 0.001). In the DAPA+VA group, these scores were lower than those in the VA group; although the differences did not reach statistical significance, DAPA treatment was associated with a trend toward reduction in the VA-induced increases in TNF-α (the most pronounced trend), IL-1β, and IL-6. The histopathological staining images (Figure 14, Figure 15, Figure 16 and Figure 17) and scoring results suggest that DAPA may suppress the inflammatory response. Overall, these findings indicate that DAPA provides a partial protective effect by attenuating VA-induced inflammation. According to Figure 15, the immunostaining intensities of TNF-α in all groups are presented. The most intense staining was observed in the VA group, while co-administration of VA and DAPA reduced the staining intensity.

## 4. Discussion

In our study, we assessed the effects of DAPA on renal tissue in VIN, as well as on inflammatory and apoptotic mediators, using histopathological analysis and serum assays. We found that VA administration in rats resulted in increased serum oxidative stress, apoptosis markers, and histopathological damage in renal tissue. We demonstrated that the co-administration of DAPA and VA attenuates VIN. This is the second study to show that DAPA attenuates VIN and that this effect is independent of hyperglycemia [40].

In this study, we provided a more detailed evaluation of the role of inflammation and showed that DAPA exerts anti-inflammatory effects in the VIN model by reducing the renal expression of pro-inflammatory cytokines, such as IL-1, IL-6, and TNF-α.

VA is the preferred agent for the treatment of infections caused by methicillin-resistant Staphylococcus aureus (MRSA) [4]. Higher target levels of VA (15–20 μg/mL) are recommended to overcome bacterial resistance and ensure an adequate response to treatment; however, this potentially increases the risk of nephrotoxicity [4,57]. Reports indicate that the incidence of VIN ranges from 5% to 35% [58]. Although the exact mechanism of VIN is not fully understood, existing animal studies suggest that VA exerts oxidative effects on the proximal renal tubules and that the use of antioxidants may mitigate the risk of VIN [9,14,46,59].

Studies have also demonstrated that VA can alter the energy-dependent renal reabsorption processes of the proximal tubule and impact mitochondrial function. This toxicity primarily arises from an increased production of reactive oxygen species (ROS) within the mitochondria, leading to mitochondrial membrane depolarization and the initiation of the caspase pathway [60,61]. Based on these animal studies, renal tubular ischemia resulting from the oxidative effects of VA is the primary identified mechanism of VIN. Protective strategies against VIN include the use of mitochondria-targeted antioxidants and cilastatin to decrease VA uptake, as well as targeting SOD to renal proximal tubule cells [60,61,62]. These strategies could potentially reduce oxidative stress and help preserve renal function during VA therapy. In our study, we evaluated the efficacy of DAPA in mitigating VIN in rats by examining the markers of oxidative stress, apoptosis, and inflammatory mediators.

SGLT2 inhibitors, including DAPA, have emerged as a novel class of antidiabetic agents with significant renoprotective properties extending beyond glycemic control [24]. These agents act by inhibiting sodium and glucose reabsorption in the renal proximal tubules, thereby promoting glucosuria and natriuresis [24,63]. This mechanism not only lowers plasma glucose levels, reducing glucotoxicity and subsequent renal injury but also confers multiple direct and indirect benefits to kidney function [63,64]. A growing body of evidence demonstrates that SGLT2 inhibitors reduce intraglomerular pressure, suppress activation of the renin–angiotensin–aldosterone system (RAAS), and enhance renal energy utilization [64]. By decreasing sodium reabsorption, SGLT2 inhibitors increase tubuloglomerular feedback, leading to afferent arteriolar constriction and a subsequent reduction in glomerular hyperfiltration—a key driver of progressive nephron injury in both diabetic and non-diabetic kidney disease [65]. Furthermore, these agents have been shown to decrease albuminuria, renal inflammation, and oxidative stress while also exerting favorable effects on blood pressure, body weight, and serum uric acid levels, all of which may contribute to their overall renoprotective profile [64,65]. Clinical trials and experimental studies have consistently demonstrated that DAPA slows the decline in GFR, reduces proteinuria, and mitigates both glomerular and tubular injury in diabetic models [25,27,29]. Notably, the renoprotective effects of DAPA have also been observed in non-diabetic settings, such as chronic kidney disease and heart failure, highlighting its broad therapeutic potential [28,30,31]. The anti-inflammatory and antioxidative properties of SGLT2 inhibitors are increasingly recognized as central to their nephroprotective mechanisms [25,26].

In diabetic rat and mouse models, DAPA slows the decline in GFR, reduces proteinuria, and limits both glomerular and tubular injury [27,29]. Additionally, it attenuates oxidative stress and inflammation, suppresses the activation of the renin–angiotensin–aldosterone system, and inhibits TGF-β-associated fibrotic pathways [25,26,29,40,64,65]. Collectively, these effects contribute significantly to the retardation of diabetic nephropathy progression and the preservation of renal function.

In nephrotoxicity models induced by agents such as VA, cisplatin, and gentamicin, increased oxidative stress is a prominent feature [2,9,60]. DAPA significantly reduces oxidative stress by decreasing lipid peroxidation products such as MDA and TOS while enhancing antioxidant defense systems, including TAS, SOD, GPx, and CAT [25,26,40]. Additionally, it prevents mitochondrial dysfunction and membrane depolarization and suppresses the activity of pro-oxidant enzymes such as MPO [60,61,66]. Through these mechanisms, DAPA effectively mitigates oxidative damage and the resulting cellular dysfunction in renal tissue [40,60,61,66].

Inflammation is another critical contributor to the pathogenesis of AKI and chronic kidney disease. DAPA exerts anti-inflammatory effects by downregulating the expression of pro-inflammatory cytokines, such as TNF-α, IL-1β, and IL-6, in renal tissue [26,67,68]. In addition, DAPA restricts the expression of inflammatory genes by inhibiting NF-κB activation [69]. Moreover, DAPA attenuates renal interstitial fibrosis through the inhibition of TGF-β-associated fibrotic signaling pathways [29]. As a result of these effects, DAPA provides histopathological protection in nephrotoxicity models (such as VA, cyclosporine, and colistin) by reducing tubular necrosis, inflammation, atrophic changes, and hyaline cast formation [34,36,40].

From a safety perspective, DAPA is generally well tolerated. The most common adverse effects are genitourinary infections, particularly in women, as well as volume depletion and hypotension due to osmotic diuresis [24]. The risk of hypoglycemia is low when used as a monotherapy but may increase when combined with insulin or sulfonylureas [24]. Rare but serious adverse events include euglycemic diabetic ketoacidosis, Fournier’s gangrene, and, infrequently, AKI or bone fractures [24]. Mild increases in LDL cholesterol and occasional electrolyte disturbances have also been reported [24].

When free radicals cause lipid peroxidation, they produce MDA, which indicates oxidative damage and results in increased membrane permeability [70]. In our study, MDA levels were significantly higher in the VA-administered group compared to the control group. This finding suggests a potential role of oxidative stress in VIN. The group that received both DAPA and VA exhibited a trend toward lower MDA levels compared to the group that received only VA. Although the VA group exhibited the highest levels of both TOS and MPO, there was no statistically significant difference observed in the co-administered DAPA with VA. Our study also found that among the antioxidant markers, which include GPx, CAT, SOD, and TAS, only the levels of SOD were significantly higher in the VA group compared to the other groups. However, SOD levels did not differ between those who administered VA alone and those who administered VA in combination with DAPA. The lack of statistically significant differences in certain parameters related to vancomycin-induced kidney injury and dapagliflozin administration has been explained in the literature by various mechanisms. The severity and timing of the experimental model may not be sufficient for some biomarkers to demonstrate detectable changes, and certain markers may only exhibit significant alterations at specific phases of injury [71,72]. In addition, due to the balance between antioxidant and oxidative stress mechanisms in renal tissue, the levels of enzymes such as GPx and CAT may be compensated by alternative pathways [73]. Since an inflammatory response is prominent in vancomycin-induced injury, marked changes are particularly observed in pro-inflammatory cytokines, while alterations in parameters such as TOS, MPO, Bax, and Bcl-2 may be less pronounced [59]. The sensitivity of the measurement methods and the heterogeneity within tissue samples may also have contributed to these findings [74]. These findings may suggest that the antioxidant effect of DAPA is less pronounced compared to other renoprotective mechanisms and that inflammatory mechanisms primarily underlie the development of kidney injury. Further research is needed to explore the underlying mechanisms and determine how DAPA can be optimized for better therapeutic outcomes.

VIN has been associated with apoptosis [60,61]. In studies exploring nephrotoxicity, the endoplasmic reticulum initiates apoptosis when calcium homeostasis is disrupted or when misfolded proteins accumulate. Apoptosis results in the loss of renal epithelial cells during kidney injury. Consequently, both caspase-dependent and -independent signaling pathways, including the mitochondrial pathway, trigger cell death [60,61]. VA induces an imbalance between ROS and antioxidants, stimulating oxygen consumption and resulting in a dose-dependent increase in intracellular ATP levels, ultimately leading to mitochondrial dysfunction and cellular apoptosis within renal cells [60]. In our study, we comprehensively evaluated the expression of key apoptotic mediators, including the pro-apoptotic proteins Bax and Casp-3, as well as the anti-apoptotic protein Bcl-2, in the context of VIN. Our results demonstrate that VA administration significantly increased Casp-3 activity, indicative of heightened apoptotic signaling in renal tissue. Notably, co-administration of DAPA with VA markedly attenuated Casp-3 activity, suggesting a potent anti-apoptotic effect. This observation is consistent with previous findings by Darwish et al., who reported that DAPA effectively reduced caspase-dependent apoptosis in a similar experimental model [40]. The anti-apoptotic properties of DAPA are increasingly recognized as central to its renoprotective profile. Recent research has shown that DAPA can alleviate endoplasmic reticulum (ER) stress and apoptosis in renal tubular epithelial cells, further supporting its cytoprotective effects [66]. In addition, studies investigating other nephroprotective agents, such as the flavonol glycoside rutin, have demonstrated that inhibition of VA-induced caspase activation is a key mechanism underlying protection against VIN [72]. Similarly, DAPA has been shown to reduce apoptosis in cyclosporine-induced nephrotoxicity, highlighting its broad-spectrum anti-apoptotic potential [34].

Our findings suggest that the concurrent administration of DAPA with VA safeguards renal cells by inhibiting VA-induced caspase activation, stabilizing the mitochondrial membrane potential, and thereby reducing subsequent apoptosis. This aligns with the accumulating evidence indicating that the renoprotective effects of DAPA are at least partly mediated by its ability to suppress apoptotic pathways [35,40]. The observed modulation of both pro- and anti-apoptotic mediators in our study reinforces the hypothesis that apoptosis reduction is a key contributor to the nephroprotective efficacy of DAPA. However, the precise molecular pathways through which DAPA exerts these protective effects remain to be fully elucidated. While our data and the existing literature highlight the central role of apoptosis inhibition, additional mechanisms—such as the attenuation of oxidative stress, improvement of mitochondrial function, and modulation of inflammatory responses—may also be involved. Further studies employing advanced molecular techniques are warranted to delineate these pathways. A deeper understanding of the mechanisms underlying DAPA’s renoprotective actions could facilitate the development of more effective therapeutic strategies for patients at risk of drug-induced nephrotoxicity.

The normal immune response to infections relies on the nuclear factor kappa-light-chain-enhancer of activated B cells (NF-κB); however, the dysregulated activation of NF-κB is a significant factor in the development of inflammatory diseases. Research indicates that NF-κB plays a critical role in the pathophysiology of kidney inflammation triggered by autoimmune processes, injury, or infection. The activation of NF-κB contributes to the pathogenesis of renal injury by promoting the release of inflammatory mediators such as TNF-α, IL-1β, and IL-6 [69]. Inflammatory cytokines are pivotal in both AKI and the progression of chronic kidney disease. During acute kidney injury, cytokines are produced by leukocytes and renal tubular cells, contributing to the initiation and exacerbation of inflammation [75]. These inflammatory mediators affect tubular epithelial cells, leading to apoptosis, necrosis, and epithelial-to-mesenchymal transition [76]. In models of ischemia, sepsis, and nephrotoxicity, the initial injury results in changes to tubular epithelial and vascular endothelial cells, which subsequently cause leukocyte infiltration and enhance the production of cytokines [77]. SGLT2 inhibitors have demonstrated efficacy in reducing inflammatory markers such as CRP, IL-6, and TNF-α in both clinical and preclinical studies [67,68]. Our research is the first to investigate the expression of the pro-inflammatory cytokines TNF-α, IL-1β, and IL-6 in the renal tissue of rats with VIN. The co-administration of DAPA with VA resulted in a reduction trend in the expression levels of all three cytokines compared to the VA-only group, with the most prominent decrease observed in TNF-α levels; however, this reduction did not reach statistical significance for any of the cytokines. This result suggests that DAPA may play a critical role in modulating the inflammatory response associated with VIN, offering potential therapeutic benefits. Further exploration is needed to fully understand the underlying mechanisms and assess the long-term implications of these findings.

In a review of biopsy-confirmed cases of VA nephrotoxicity, acute tubular necrosis and tubulointerstitial nephritis were identified as the predominant manifestations of VIN [78]. The administration of VA has been associated with significant histopathological damage in rat kidney tissues. Limited studies have documented a range of renal effects at elevated doses, including glomerular destruction, proximal tubule necrosis, and mild histological alterations [79,80]. Histological examinations under light microscopy of kidney tissue from rats administered VA revealed tubular necrosis, tubular dilatation, tubular desquamation, renal parenchymal congestion, and inflammation. In the group co-administering DAPA with VA, we observed improvements in these histopathological findings. Numerous studies have documented the renal protective effects of SGLT2 inhibitors based on experimental research [32,34,36]. SGLT2 inhibitors provide renal protection by reducing oxidative stress, inflammation, fibrosis, and tubuloglomerular feedback, as demonstrated in animal studies [81,82,83]. Our study reveals the positive effects of DAPA on the oxidative stress response, inflammation, and apoptosis within renal tissue. These mechanisms suggest that SGLT2 inhibitors may play a critical role in mitigating kidney damage in various pathological conditions. Further investigation into the biochemical pathways involved could elucidate the full extent of their protective effects and guide future therapeutic strategies in renal disease management.

The study has several limitations. First, the reliance on an animal model may not accurately reflect human physiology and can limit the applicability of findings to clinical settings. The short duration of treatment may not account for the long-term effects that could be observed in patients. Additionally, the focus on specific inflammatory and apoptotic markers could overlook other relevant mechanisms involved in renal injury and recovery. The histopathological evaluation of renal tissue was performed using light microscopy and scored subjectively by a blinded pathologist. While this approach is standard, it may introduce variability and potential bias in the interpretation of results. Advanced imaging techniques or molecular analyses could provide more objective and detailed insights. Importantly, it should be emphasized that this study is an experimental investigation, and the findings cannot be directly generalized to clinical practice. Translational gaps between animal models and human physiology remain, and the potential nephroprotective effects of dapagliflozin in vancomycin-induced nephrotoxicity must be corroborated in well-designed clinical trials before any recommendations for human use can be made.

In conclusion, while the study provides promising evidence for the nephroprotective effects of DAPA in a rat model of VIN, its limitations highlight the need for further research. Future studies should focus on longer durations, larger sample sizes, dose-response analyses, the exploration of additional biomarkers, and clinical trials to better understand the therapeutic potential of DAPA in human nephrotoxicity.

## 5. Conclusions

This study investigated the potential protective effects of DAPA against VIN. The findings suggest that DAPA may protect renal tissue by reducing oxidative stress, inflammation, and apoptosis. VA administration increased oxidative stress markers (e.g., MDA) and apoptotic indicators (e.g., Casp-3), while co-administration with DAPA significantly mitigated these effects and was associated with a trend toward a reduction in MDA levels.

Histopathological analysis shows that DAPA alleviated tubular necrosis, inflammation, and other signs of kidney damage caused by VA. Additionally, our findings demonstrate that DAPA administration was associated with a trend toward the attenuation of the VIN-induced increase in these inflammatory cytokines, with the most prominent reduction observed in TNF-α levels. These results indicate that DAPA may exert nephroprotective effects against VIN through mechanisms involving the reduction of oxidative stress, inflammation, and apoptosis. However, further long-term, large-scale clinical studies are required to confirm these findings and translate them into clinical practice.

## Figures and Tables

**Table 1:** Reactive oxygen radicals, apoptotic mediators, antioxidant system parameters, and renal function test results among rat groups.

Mediator	Control	DAPA	VA	VA+DAPA	*p*
Urea	64.8 ± 5.6 ^a,b^	71.1 ± 5.5 ^a,d^	191.2 ± 75.1 ^b,d,f^	73.4 ± 7.1 ^f^	<0.001
Creatinine	0.75 ± 0.06 ^c^	0.78 ± 0.09 ^d^	3.67 ± 2.4 ^c,d,f^	0.86 ± 0.11 ^f^	<0.001
MDA	6.28 ± 2.2 ^b^	7.47 ± 1.8	11.0 ± 5.2 ^b,f^	6.43 ± 1.2 ^f^	0.016
TOS	4.09 ± 2.4	5.60 ± 3.9	5.94 ± 4.5	4.20 ± 3.9	0.609
MPO	66.4 ± 9.3	67.1 ± 9.7	68.6 ± 10.9	58.1 ± 9.0	0.256
GPx	597.5 ± 52.9	621.6 ± 103.9	606.4 ± 154.3	593.2 ± 45.7	0.809
CAT	56.2 ± 14.9	56.7 ± 14.5	57.1 ± 6.9	49.5 ± 6.3	0.311
SOD	224.9 ± 17.2 ^b,c^	236.6 ± 18.3	278.7 ± 46.5 ^b^	278.9 ± 41.7 ^c^	0.004
TAS	1.41 ± 0.08	1.35 ± 0.07 ^d^	1.61 ± 0.19 ^d^	1.39 ± 0.16	0.014
Bax	7.47 ± 1.40	8.91 ± 1.4	8.02 ± 1.2	8.87 ± 1.3	0.130
Casp-3	2.33 ± 1.2	2.88 ± 1.9	7.53 ± 8.3 ^f^	1.89 ± 0.8 ^f^	0.026
Bcl-2	6.50 ± 1.0	6.71 ± 0.9	6.32 ± 0.8	6.65 ± 1.2	0.873

^a^ Control-DAPA, ^b^ control-VA, ^c^ control-VA+DAPA, ^d^ DAPA-VA, ^f^ VA-VA+DAPA. Data are expressed as mean ± standard deviation. DAPA, dapagliflozin; VA, vancomycin; MDA, malondialdehyde; TOS, total oxidant status; Bcl-2, B-cell lymphoma-2; Bax, Bcl-2-associated X protein; Casp-3, caspase-3; TAS, total antioxidant status; GPx, glutathione peroxidase; CAT, catalase; SOD, superoxide dismutase; MPO, myeloperoxidase.

**Table 2:** Renal histopathological findings in different rat groups.

Parameters		Control	DAPA	VA	VA+DAPA	*p*
Tubular dilatation	Normal	5 ^a^ (71.4)	6 ^a^ (85.7)	-	5 ^a^ (71.4)	<0.001
	Light	2 ^a^ (28.6)	1 ^a^ (14.3)	-	2 ^a^ (28.6)	
	Moderate	-	-	1 ^a^ (14.3)	-	
	Severe	-	-	6 ^b^ (85.7)	-	
Tubular vacuolization	Normal	4 ^a^ (57.1)	2 ^a,b^ (28.6)	-	1 ^a,b^ (14.3)	<0.001
	Light	3 ^a,b,c^ (42.9)	5 ^c^ (71.4)	-	4 ^a,c^ (57.1)	
	Moderate	-	-	-	2 ^a^ (28.6)	
	Severe	-	-	7 ^b^ (100)	-	
Hyaline cast	Normal	1 ^a^ (14.3)	-	-	-	<0.001
	Light	6 ^a^ (85.7)	7 ^a^ (100)	-	5 ^a^ (71.4)	
	Moderate	-		3 ^a^ (42.9)	2 ^a^ (28.6)	
	Severe	-	-	4 ^b^ (57.1)	-	
Tubular necrosis	Normal	5 ^a^ (71.4)	7 ^a^ (100)	-	4 ^a^ (57.1)	<0.001
	Light	2 ^a^ (28.6)	-	-	2 ^a^ (28.6)	
	Moderate	-	-	1 ^a^ (14.3)	1 (14.3)	
	Severe	-	-	6 ^b^ (85.7)	-	
Tubular atrophy	Normal	6 ^a,b^ (85.7)	7 ^b^ (100)	-	3 ^a,c^ (42.9)	<0.001
	Light	1 ^a^ (14.3)	-	-	3 ^a^ (42.9)	
	Moderate	-	-	2 ^a^ (28.6)	1 ^a^ (14.3)	
	Severe	-	-	5 ^b^ (71,4)		
Interstitial edema	Normal	5 ^a^ (71.4)	2 ^a,b^ (28.6)	-	2 ^a,b^ (28.6)	<0.001
	Light	2 ^a,b^(28.6)	5 ^b^ (71.4)	-	3 ^a,b^ (42.9)	
	Moderate	-		1 ^a^ (14.3)	2 ^a^ (28.6)	
	Severe	-	-	6 ^b^ (85.7)	-	
Tubular inflammation	Normal	7 ^a^ (100)	7 ^a^ (100)	-	6 ^a^ (85.7)	<0.001
	Light	-	-	-	-	
	Moderate	-	-	1 ^a^ (14.3)	1 ^a^ (14.3)	
	Severe	-	-	6 ^b^ (85.7)	-	
Mononuclear cells in the medulla	Normal	6 ^a^ (85.7)	7 ^a^ (100)	-	4 ^a^ (57.1)	<0.001
	Light	1 ^a^ (14.3)	-	-	2 ^a^ (28.6)	
	Moderate	-	-	-	-	
	Severe	-	-	7 ^b^ (100)	1 ^a^ (14.3)	
Medullary hemorrhage	Normal	7 ^a^ (100)	7 ^a^ (100)	-	6 ^a^ (85.7)	<0.001
	Light	-	-	-	-	
	Moderate	-	-	1 ^a^ (14.3)	1 ^a^ (14.3)	
	Severe	-	-	6 ^b^ (85.7)	-	
Necrotic area in cortex	Normal	7 ^a^ (100)	7 ^a^ (100)	-	6 ^a^ (85.7)	<0.001
	Light	-	-	-	-	
	Moderate	-	-	4 ^b^ (57.1)	1 ^a,b^ (14.3)	
	Severe	-	-	3 ^a^ (42.9)	-	
Tubular desquamation	Normal	5 ^a,b^(71.4)	7 ^b^ (100)	-	3 ^a,c^ (42.9)	<0.001
	Light	2 ^a^ (28.6)	-	-	3 ^a^ (42.9)	
	Moderate	-	-	-	1 ^a^ (14.3)	
	Severe	-	-	7 ^b^ (100)	-	

Values are presented as *n* (%). Superscript letters indicate whether there is statistically significant difference between proportions. Groups sharing same letter do not differ significantly, while groups with different letters are significantly different (*p* < 0.05).

**Table 3:** Renal tissue immunostaining intensities for TNF-α, IL-1β, and IL-6 in different rat groups.

Parameter	Control	DAPA	VA	VA+DAPA	*p*
TNF-*α*	2.00 ± 0.0 ^a,b,c^	3.00 ± 0.0 ^a,d^	12.43 ± 0.53 ^b,d^	3.43 ± 0.53 ^c^	<0.001
IL-1β	1.71 ± 0.48 ^b,c^	2.71 ± 0.48 ^d^	12.29 ± 1.25 ^b,d^	5.57 ± 1.39 ^c^	<0.001
IL-6	1.43 ± 0.53 ^b,c^	2.29 ± 0.49 ^d^	12.00 ± 0.82 ^b,d^	6.00 ± 0.00 ^c^	<0.001

Data are expressed as mean ± standard deviation, ^a,b,c,d,e,f^, *p* < 0.05. ^a^ Control-DAPA, ^b^ Control-VA, ^c^ Control-VA+DAPA, ^d^ DAPA-VA, ^e^ DAPA-VA+DAPA, ^f^ VA-VA+DAPA.

## Data Availability

The dataset is available upon request from the authors.

## Abbreviations

The following abbreviations are used in this manuscript:

Bcl-2	B-cell lymphoma-2
Bax	Bcl-2-associated X protein
CAT	Catalase
Casp-3	Caspase-3
DAPA	Dapagliflozin
GPx	Glutathione peroxidase
Il-6	Interleukin-6
IL-1β	Interleukin-1 βeta
MPO	Myeloperoxidase
MDA	Malondialdehyde
SGLT2	Sodium–glucose co-transporter
SOD	Superoxide dismutase
TNF-α	Tumor necrosis factor-alpha
TOS	Total oxidant status
TAS	Total antioxidant status
VA	Vancomycin
VIN	Vancomycin-associated nephrotoxicity
